# Effect of Medicaid accountable care on preventable emergency department and hospital admissions: rural-urban heterogeneity

**DOI:** 10.3389/frhs.2025.1475140

**Published:** 2025-06-26

**Authors:** Jangho Yoon, Seungbeen Ghim, Jeff Luck

**Affiliations:** ^1^ Department of Preventive Medicine & Biostatistics, F. Edward Hebert School of Medicine, The Uniformed Services University of the Health Sciences, Bethesda, MD, United States; ^2^ Henry M. Jackson Foundation for the Advancement of Military Medicine, Inc, Bethesda, MD, United States; ^3^ College of Health, Oregon State University, Corvallis, OR, United States

**Keywords:** coordinated care organization, accountable care, Medicaid, preventable ED admission, preventable hospital admission, Oregon, USA

## Abstract

**Background:**

Accountable care organizations provide a framework for collaboration among providers and payers to improve patients’ health and care experiences while reducing costs. However, there is limited research on the realization of these benefits for low-income individuals across varying degrees of rurality. This study examined the heterogeneous impact of Coordinated Care Organizations (CCOs), an accountable care model implemented in Oregon Medicaid, on preventable emergency department (ED) and hospital admissions by rurality of residence.

**Methods:**

Using person-month panel data on 131,246 adults aged 18–64 continuously enrolled in Oregon Medicaid between 2011 and 2015, we employed a doubly-robust difference-in-differences approach to isolate the impacts of the CCO model on the number of ED visits and the probability of hospital admissions, separately for all-cause and preventable admissions.

**Results:**

The CCO model was associated with reductions of 25 all-cause ED visits and 22 preventable ED visits per 1,000 persons per month during the first three years. Significant decreases in all-cause and preventable ED visits were observed across different levels of rurality. However, the magnitude of these reductions decreased almost monotonically as rurality increased from urban to small/isolated rural areas. On average, the CCO model was associated with significant declines in preventable ED visits by 18, 9, and 5 visits per 1,000 persons per month among urban, large rural, and small/isolated rural residents, respectively. No statistically discernable relationship was found for hospital admissions.

**Conclusions:**

The CCO model led to significant overall reductions in preventable ED visits. However, this beneficial effect may diminish with increased rurality.

## Introduction

Accountable care organizations (ACOs)—provider networks that form voluntarily to coordinate care and accept collective responsibility for the quality and cost of care (1)—provide a framework within which providers and payers can collaborate to improve patients’ health status and care experience while reducing costs. While ACOs have been growing fast for Medicare beneficiaries and commercially insured populations, state Medicaid programs have also been increasingly adopting comparable approaches to payment and care delivery (2–4). Currently, 14 states are implementing ACO models for their Medicaid enrollees (5). Although Medicaid ACOs differ in organization and structure across states (4), they share common goals of keeping patients healthy, improving quality, controlling costs by aligning provider incentives to value instead of volume, coordinating care, and reducing inappropriate utilization (6).

Oregon's Medicaid program, Oregon Health Plan (OHP), has pioneered innovative ways to deliver health care services to low-income and disabled individuals. In August 2012, OHP started to enroll its Medicaid beneficiaries in Coordinated Care Organizations (CCOs), Oregon's version of accountable care (7, 8). Most CCOs became operational in January 2013 (7, 8), and by 2016, over 90% of OHP beneficiaries were enrolled in CCOs (9).

While CCOs are consistent with the typical ACO delivery concept, such as shared savings and performance standards, they are also unique in that CCOs are geographically defined networks of health care providers, required to integrate medical and behavioral health care, assigned Medicaid beneficiaries automatically by residential ZIP codes, and receive risk-adjusted global payments (7). CCOs’ self-reported performance data show beneficial impacts on most performance domains. For example, from 2011 to 2018, avoidable ED visits dropped significantly from 14.2 to 6.3 visits per 1,000 member months, and all-cause hospital readmissions declined slightly from 12.9% to 11.1% of all discharges (10). McConnell et al. (11) find a statistically significant increase in inpatient days during the second year of CCO implementation. Most prior studies report positive overall impacts of the CCO model, including declines in ED visits among CCO enrollees over the two years of early implementation (11, 12) and improvement in access to prenatal care, neonatal outcomes, and reproductive health outcomes among women of reproductive age (7, 13–17).

This study contributes to the literature by examining the heterogeneous impacts of the CCO model on preventable ED visits and hospital admissions by rurality/urbanicity of residence among adult Oregon Medicaid beneficiaries. Rural residents deserve particular focus due to substantial disparities in health and access to health care compared to their urban counterparts (18, 19). These disparities stem from structural barriers, including provider shortages, longer travel distances, and limited availability of primary and urgent care services (19). As a result, rural residents often have fewer alternatives to EDs for addressing urgent health needs, which may lead to a greater reliance on EDs even for conditions that could be prevented or treated in outpatient settings (20). The scarcity of critical resources, such as health information technology, further limits the capacity of rural health systems to support integrated care models, presenting seriously challenges rural ACOs (21–24). Implementing innovative delivery models like CCOs can be resource-intensive. However, rural areas often face financial constraints and lack the robust provider networks needed to fully support such system transformation (25). Even so, some ACOs in rural areas large enough to have hospitals may opt to repurpose and absorb resources to transform existing hospitals into community health hubs, which might produce positive patient outcomes (21). This situation may disproportionately benefit patients in large rural areas more than those in small/isolated rural areas. Therefore, we hypothesize that the beneficial impact of the CCO model on preventable ED visits and hospital admissions diminishes with increased rurality of residence. CCOs are incentivized to avoid preventable ED utilization through pre-established performance standards. However, preventable hospitalizations were not included as part of the performance standards. Therefore, we hypothesize that the effect of CCOs on preventable hospital admissions, if any, is smaller than the effect on preventable ED visits.

## Methods

### Data sources and sample

We retrieved Medicaid eligibility and claims data from the Oregon Medicaid Management Information System maintained by the Oregon Health Authority Office of Health Analytics, which provided information on Medicaid enrollment, demographics, residential ZIP codes, and medical claims. The main Medicaid data files were augmented with the CCO enrollment file and Rural–Urban Commuting Area Codes (RUCAs) to identify rural residential status at the ZIP code or census tract level (26).

We created a person-month panel data set with up to 57 monthly observations per person. The original data included about 53.6 million person-months on 1,023,032 individuals aged 18–64 ever enrolled in Oregon Medicaid between January 1, 2011 and September 30, 2015. Because Medicaid enrollment can be endogenous, that is, changing month-to-month as eligibility criteria change, we restricted analysis to beneficiaries continuously enrolled in Medicaid—defined as enrollment for at least 80 percent of the six-year study period. The final analytic sample included 7,473,101 person-month observations on 131,246 adult Medicaid beneficiaries aged 18–64: approximately 79 percent of the sample (103,632 persons) were enrolled in CCOs, and the remaining 21 percent (27,614 persons) were never enrolled in a CCO during the study period.

CCO enrollment is mandatory for most, but specific subgroups are exempt, including persons dually eligible for Medicare and Medicaid; pregnant women in their third trimester at enrollment; persons under 19 years of age placed in adoptive or foster care out of state; persons under 18 years of age who are medically fragile and have special health care needs; persons receiving medical home-care services; persons with other primary medical insurance coverage; persons living in areas without a CCO; American Indian and Alaska Native (AIAN) beneficiaries; and noncitizens eligible for labor and delivery services and emergency treatment services (27). Supplementary Table S1 shows that both CCO and non-CCO enrollees were found in all the Medicaid program categories despite some compositional differences. For example, compared to non-CCO enrollees, CCO enrollees were more likely to receive the Temporary Assistance for Needy Family (TANF) and expansion Medicaid program codes but less likely to receive dual eligibility and other program codes. Otherwise, enrollees in both groups were similar compositionally.

### Variables

ED visit outcomes include the number of ED visits, separately for *all-cause* and *preventable visit*s. Following Hennessy et al. (28), we defined an all-cause ED visit as a Medicaid claim flagged as an ED episode, or having a procedure code for ED services (99281, 99282, 99283, 99284, 99285), other emergency services (99288), or critical care services (99291, 99292). A preventable ED visit was identified based on primary to fifth ICD-9 diagnosis codes and the ED use profiling algorithm developed by Billings et al. (29). This algorithm classifies ED visits according to nine diagnosis categories. A preventable ED visit was defined as an ED visit involving diagnoses for non-emergent conditions, conditions that are emergent but treatable in primary care settings, or conditions that are emergent and require ED care but are preventable. Frequencies of all-cause and preventable ED visits are reported in Supplementary Table S2.

Hospital admission outcomes were constructed as binary variables of *all-cause hospital admission* and *preventable hospital admission*. All-cause admission was coded as 1 if a Medicaid claim was flagged as an inpatient claim. We used the Prevention Quality Indicators (PQIs) to identify preventable admissions for acute and chronic ambulatory care sensitive conditions (30, 31).

The CCO group indicator identifies Medicaid beneficiaries enrolled in a CCO during the post-CCO period. The post-CCO period variable indicates the years 2013–2015, the period during which the CCO model had been implemented. The interaction term of the CCO group and post-CCO period indicators is the main variable of interest, discussed below. The rurality of residence location was quantified by a 3-level variable based on RUCAs (26). Other observed covariates included race and Latino/Hispanic ethnicity, rurality of residence, age, and sex.

### Statistical analyses

We employed a doubly-robust difference-in-differences (DID) approach, capitalizing on the fact that only Medicaid beneficiaries enrolled in CCOs during the post-CCO era were impacted by CCO implementation, while other beneficiaries in traditional fee-for-service Medicaid (reference group) were not. With a multivariate DID regression model, we sought to minimize bias from both selection on observables and unobservables.

For the count ED visit outcomes, we first estimated the following fixed-effects negative binomial regression model:

(1) $$\mu_{it}=exp(\beta_{cp}cco_{i}\times p_{t}+\beta_{p}p_{t}+x_{it}^{\prime }\beta_{x}+\beta_{t}t_{t}+\beta_{ct}cco_{i}\times t_{t}+m_{t}^{\prime }\beta_{m}+\alpha_{i}+\varepsilon_{it})$$

Here, μ is the expected number of ED visits (separately for all-cause and preventable visits); *i* and *t* subscripts denote person and month, respectively; the β’s are the coefficients to be estimated; and ε is the error term. We estimated a conditional fixed-effect logit model for the binary hospitalization outcomes (h) as following:

(2) $$Pr(h_{it}>0)=\Lambda (\beta_{cp}cco_{i}\times p_{t}+\beta_{p}p_{t}+x_{it}^{\prime }\beta_{x}+\beta_{t}t_{t}+\beta_{ct}cco_{i}\times t_{t}+m_{t}^{\prime }\beta_{m}+\alpha_{i})$$

where Λ is the cumulative logistic distribution.

The interaction term (cco×p) of the CCO group (cco) and post-CCO period (p) indicators is of primary interest as its coefficient might indicate the additional change that the CCO model had on Medicaid beneficiaries continuously enrolled in CCOs vs. the reference population of those continuously enrolled in the traditional Medicaid program. For example, a negative and significant estimate of $\beta_{cp}$ in Equations 1, 2 would indicate that the CCO model was associated with a reduced number of ED visits and a reduced probability of hospital admission, respectively, in any given month. The vector $x^{\prime }$ includes time-varying covariates, age, and rurality categories. A linear month time trend (t) and its interaction with the CCO group indicator (cco×t) together capture time trends specific to the CCO and reference groups, easing potential concern that pre-existing trends in the outcomes might not be completely ruled out. A vector of month dummies $\left(m^{\prime }\right)$ was included to capture seasonality parametrically. The unobserved person heterogeneity term ($\alpha_{i}$) was included to relax the assumption in the prior studies that unobserved person characteristics were uncorrelated with CCO enrollment.

We then augmented the reference model specifications as follows with triple interaction terms of the CCO group, post-CCO period, and rurality indicators to examine heterogeneous effects of CCO implementation by rurality of residence:

(3) $$\begin{array}{r} \mu_{it}=exp(\alpha_{i}+\beta_{cp}cco_{i}\times p_{t}+\beta_{cplr}cco_{i}\times p_{t}\times lr_{it}+\beta_{cpsr}cco_{i}\times p_{t}\times sr_{it}\\ +\beta_{p}p_{t}+x_{it}^{\prime }\beta_{x}+\beta_{t}t_{t}+\beta_{ct}cco_{i}\times t_{t}+m_{t}^{\prime }\beta_{m}+\alpha_{i}+\varepsilon_{it}) \end{array}$$

(4) $$Pr(h_{it}>0)=\Lambda (\alpha_{i}+\beta_{cp}cco_{i}\times p_{t}+\beta_{cplr}cco_{i}\times p_{t}\times lr_{it}+\beta_{cpsr}cco_{i}\times p_{t}\times sr_{it}+\beta_{p}p_{t}+x_{it}^{\prime }\beta_{x}+\beta_{t}t_{t}+\beta_{ct}cco_{i}\times t_{t}+m_{t}^{\prime }\beta_{m}+\alpha_{i})$$

where $cco_{i}\times p_{t}\times lr_{it}$ and $cco_{i}\times p_{t}\times sr_{it}$ are the triple interaction terms for residence location in large rural areas ($lr_{it}$) and small/isolated rural areas ($sr_{it}$), respectively. The coefficient on the interaction term $cco_{i}\times p_{t}$ ($\beta_{cp}$) now captures the effect of CCO for those in the reference residential location (i.e., urban CCO enrollees). The coefficients on the triple interaction terms, $\beta_{cplr}$ and $\beta_{cpsr}$, represent the differences for large rural and small/isolated rural areas, respectively, relative to urban areas. We computed marginal effects separately for each rurality/urbanicity group as a linear combination of coefficients.

The accuracy of our quasi-experimental approach relies on the assumption that trends in the outcomes would have been the same between the CCO and control groups had the CCO model not been implemented. The so-called conditional parallel trend condition was satisfied in our data. As shown in Supplementary Figure S1, pre-CCO trends in the outcomes from 2011 to 2012 appear similar for both population groups. We also carried out regression-based falsification analyses to test for pre-existing time trends in the outcomes on pre-CCO data with pseudo-policy period indicators (e.g., assuming the CCO model started in January 2011 or January 2012). The coefficient on the interaction term was always insignificant, validating the DID assumption, shown in Supplementary Table S3.

The fixed-effects negative binomial regression models were estimated via a hybrid method that runs random-effects negative binomial regression on time-demeaned regressors (32). Because coefficients in non-linear models (especially those on interaction terms) are often misleading (33, 34), we obtained the so-called average marginal effect via the finite-difference method, which measures an average difference in the expected number of ED visits between CCO and non-CCO enrollees. For the hospitalization outcomes, we obtained marginal effects via fixed-effects linear probability models for which all predicted probabilities were within the unit interval, ranging from 0.04 to 0.11.

All estimates were inverse-probability weighted, so our estimates may have doubly-robust property. As shown in Supplementary Table S4, we estimated a propensity-score model with a relaxed functional form (i.e., interaction terms). All statistical analyses were conducted using Stata version 18 (StataCorp LLC, College Station, TX).

## Results

Table 1 provides pretreatment means and standard deviation of the variables, unweighted and inverse-probability weighted. Approximately 79% of the unweighted sample were CCO enrollees over the study period, and 58% of the observations were for the post-CCO period. The average age was 40.8, and females comprised 63.3% of the sample. The majority of the sample was White (77.7%) followed by Black (4.55%), Asian (2.35%), American Indian/Alaskan Native (2.07%), and Native Hawaiian/Pacific Islander (0.24%). Eleven percent of the sample was Hispanic. The majority (81.7%) of the sample lived in urban areas. Large and small rural residents comprised 14.4% and 3.9%, respectively. CCO enrollees were slightly younger and more likely to be female than non-CCO enrollees. CCO enrollees were more likely to be White, Black, or Asian but less likely to be American Indian and Alaskan Native, Native Hawaiian and Pacific Islander, or Hispanic. CCO enrollees were less likely to live in rural areas than non-CCO Medicaid beneficiaries, as expected. Notwithstanding, the baseline characteristics of CCO and non-CCO enrollees overall do not appear to be substantially different from each other, which is even more likely for inverse-probability weighted means.

**Table 1:** Descriptive characteristics of the entire sample and pre-CCO period sample.

Variables	Unweighted			Inverse probability weighted		
	Entire sample (%)	Subsamples		Entire sample (%)	Subsamples	
		CCO enrollees (%)	Non-CCO Medicaid beneficiaries (%)		CCO enrollees (%)	Non-CCO Medicaid beneficiaries (%)
*n*	7,472,359	2,486,564	662,671	7,472,359	2,486,564	662,671
Key explanatory variables						
CCO^a^ enrollment	79.0	100	0	49.8	100	0
Post period	57.9	0	0	57.9	0	0
CCO enrollment X Post period	45.7	0	0	28.8	0	0
Covariates						
Age [mean (s.d./s.e.)]	40.8 (0.0045)	38.7 (0.0078)	42.6 (0.014)	40.3 (0.0072)	38.7 (0.0078)	39.2 (0.021)
Female	63.3	65.2	56.1	63.4	65.18	61.7
Race/ethnicity						
White	77.7	79.9	69.5	80.7	79.9	81.4
Black	4.55	5.06	2.63	5.12	5.06	5.19
AI/AN^b^	2.07	1.62	3.77	1.70	1.62	1.77
Asian	2.35	2.64	1.25	2.68	2.64	2.72
NHPI^c^	0.24	0.20	0.36	0.20	0.20	0.20
Unknown/missing	13.1	10.6	22.5	9.65	10.6	8.68
Hispanic	11.0	7.13	25.7	6.98	7.13	6.82
Rurality of residence						
Urban areas	81.7	82.5	78.5	81.9	82.5	81.2
Large rural City/Town	14.4	13.9	16.2	14.5	13.9	15.2
Small/isolated small Rural Town	3.93	3.54	5.32	3.61	3.54	3.66
Outcomes						
# all-cause ED visits [mean (s.d./s.e.)]	0.072 (0.0001)	0.082 (0.0002)	0.036 (0.0003)	0.071 (0.0002)	0.082 (0.0002)	0.060 (0.0007)
# preventable ED visits [mean (s.d./s.e.)]	0.062 (0.0001)	0.069 (0.0002)	0.030 (0.0003)	0.061 (0.0002)	0.069 (0.0002)	0.051 (0.0006)
All-cause hospital admission	1.60	1.64	1.56	1.89	1.64	2.21
Preventable hospital admission	0.80	0.72	0.75	1.01	0.72	1.17

^a^ CCO, Coordinated Care Organization.

^b^ AI/AN, American Indian/Alaskan Native.

^c^ NHPI, Native Hawaiian/Pacific Islander.

For the entire sample, the average number of all-cause ED visits was 0.072 per person per month (or 72 visits per 1,000 persons per month); preventable ED visits averaged 0.062 per person per month; and about 1.6% of the entire observations had all-cause hospital admissions, while 0.8% had preventable hospital admissions. Overall, CCO enrollees were more likely than non-CCO enrollees to have ED and hospital admissions.

Table 2 presents coefficients from the fixed-effects DID models that investigated the overall effects of the CCO model. The coefficient on the interaction term of CCO-enrollee and post-CCO period indicators is of primary interest. It was negative and statistically significant for both any-cause and preventable ED visits, which might suggest a decrease in ED visits following the implementation of the CCO model. In comparison, although negative, the coefficients on the main interaction term for the hospital admission outcomes were statistically insignificant and small in magnitude. Both the number of ED visits and the probability of hospital admissions decreased as the degree of rurality increased: compared to urban residents, rural residents, especially in small/isolated rural areas, were less likely to visit EDs and be admitted to hospitals. The coefficients on the linear time trend and its interaction with the CCO enrollee indicator were jointly significant, which indicates group-specific time trends in the outcomes. Specifically, the time trend coefficient indicated an overall decline in ED visits for non-CCO enrollees, whereas CCO enrollees experienced an overall increase during the study period. For hospital admissions, there was no significant time trend among non-CCO enrollees, but CCO enrollees showed a significant upward trend over time.

**Table 2:** Effects of CCO on ED visits and hospital admissions: coefficients from fixed-effects negative binomial and logit models.

Variables	Number of ED^b^ visits		Hospital admission	
	All-cause	Preventable	All-cause	Preventable
CCO^a^ × post	‒0.3123***	‒0.2963***	‒0.0422	‒0.0603
	(0.0387)	(0.0413)	(0.0423)	(0.0553)
Post	0.1272***	0.1193***	0.0013	‒0.0245
	(0.0328)	(0.0351)	(0.0405)	(0.0521)
Age	‒0.0044	0.0041	‒0.0020	0.0042
	(0.0106)	(0.0111)	(0.0175)	(0.0237)
Rurality (reference: urban)				
Large rural	‒0.4411***	‒0.4566***	‒0.1859***	‒0.1885**
	(0.0721)	(0.0672)	(0.0403)	(0.0585)
Small rural	‒0.5759***	‒0.5749***	‒0.2620***	‒0.3133***
	(0.0848)	(0.0899)	(0.0542)	(0.0815)
Time trend	‒0.0058***	‒0.0056***	‒0.0019	0.0025
	(0.0012)	(0.0013)	(0.0020)	(0.0026)
CCO × time trend	0.0138***	0.0135***	0.0028*	0.0068***
	(0.0010)	(0.0011)	(0.0014)	(0.0018)
α	8.593 (0.194)	8.891 (0.236)		
Likelihood test of overdispersion $\left(H_{0}:\;\alpha =0\right)$	$\chi^{2}$=3.4e + 05***	$\chi^{2}$=2.7e + 05***		
*n*	7,473,044	7,473,044	4,231,805	4,221,758

^a^ CCO = Coordinated Care Organization.

^b^ ED = Emergency department. Fixed-effects negative binomial models were used for the count ED visit outcomes. Conditional fixed-effects logit models were estimated for the binary hospital admission outcomes. Estimates are inverse-probability weighted. Standard errors in parentheses were corrected for clustering on individuals. All models included month dummies.

* *p* < .05.

** *p* < .01.

*** *p* < .001.

**Figure 1:**
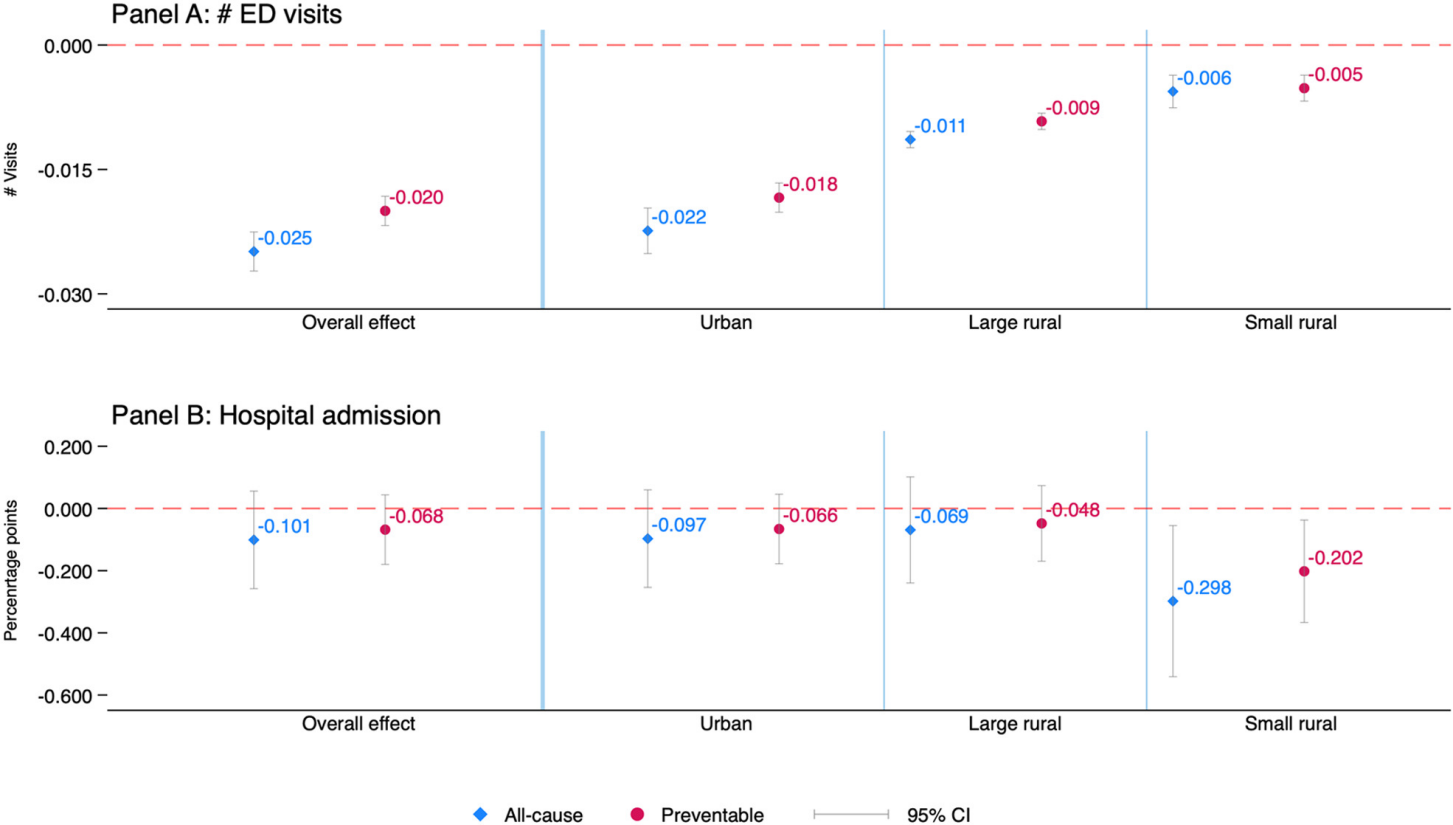
Effects of CCO on ED visits and hospital admissions: (average) marginal effects. Results are from the augmented model that included additional triple interaction terms of the CCO group indicator, post-CCO period indicator, and rurality/urbanicity categories. Average marginal effects (bootstrapped standard errors) are reported for the ED visit outcomes calculated as average pre-post changes in predicted counts of ED visits among CCO enrollees as compared to average pre-post changes in predicted counts of ED visits among non-CCO individuals, separately for each rurality/urbanicity. Reported for the hospital admission outcomes are marginal effects (and cluster-robust standard errors corrected for intraclass correlation within individuals), obtained directly from the fixed-effects linear probability model. We computed the marginal effect presented for each rurality/urbanicity subgroups as the linear combination of coefficients for the reference group (urban residents) plus the interaction term coefficient for that rurality/urbanicity category. All estimates are inverse-probability weighted. Full results are provided in Supplementary Table S5, available as a supplement to the online version of this article.

Figure 1 depicts average marginal effects that measure the magnitude of the discovered relationships. We focus on main results and Supplementary Table S5 presents the full results. The CCO model overall was associated with decreases in the number of all-cause ED visits and preventable ED visits by 0.025 and 0.020 per person per month, respectively. The discovered relationships appear substantively significant as well in that among CCO enrollees, the 0.020 decrease in preventable (all-cause) ED visits represents ∼31% of the pre-CCO average of all-cause ED visits (0.082 per person per month), and the 0.020 decrease in preventable ED visits represents ∼29% of the pre-CCO average of preventable ED visits (0.069 per person per month).

The CCO model was significantly associated with decreases in all-cause and preventable ED visits for CCO enrollees throughout the rurality/urbanicity of residence location. Notwithstanding, the magnitude of the relationship decreased almost monotonically and statistically significantly as the level of rurality increased from urban to small/isolated rural areas. In particular, the CCO model, on average, was associated with significant declines in preventable ED visits by 0.018, 0.009, and 0.005 visits per person per month among urban, large rural, and small/isolated rural residents, respectively.

The marginal effect of the CCO model on hospitalization was always negative, but overall, it was not statistically discernable. The CCO model was significantly associated with 0.30 and 0.20 percentage-point declines in the probability of all-cause and preventable hospital admission per person per month, respectively, among those living in small/isolated rural areas. However, the statistical significance disappeared in robust checks, as shown below.

### Robustness checks

In our main analysis, continuous enrollment in Medicaid was defined as being enrolled in Medicaid during at least 80 percent of the 2011–2015 study period. We tested whether our results were sensitive to the most stringent definition of 100 percent enrollment in Medicaid during the entire study period and to a generous threshold of ≥60 percent. We re-estimated all the models, not adjusting estimates with inverse probability weights. Our findings would be more credible if they were robust to the observed baseline differences between CCO and non-CCO enrollees. So, we tested whether specific Medicaid program categories drove our results by dropping individuals in the major Medicaid categories with unbalanced composition, one by one, including TANF, dually-eligible beneficiaries, and the expansion population. As shown in Supplementary Table S6, our main findings remained robust for the ED visit outcomes. However, the statistically significant estimates found for some hospital admission outcome models were not always robust.

## Discussion

Several states have adopted ACO models as an integrated health care delivery approach for their Medicaid beneficiaries (3). This study examined the impacts of CCOs, an ACO model implemented in Oregon Medicaid, on preventable ED and hospital admissions with focus on rural-urban heterogeneity. Our findings indicate that the CCO model significantly reduced ED visits, largely through preventable ED visits over the first three years of implementation. Although not directly comparable to other estimates from the literature, our estimates are qualitatively consistent with an 11% decrease in the probability of all-cause ED visits in two years of CCO implementation reported in prior analysis (11). Our findings are also consistent with the broader literature evaluating Medicaid ACO performance, which documents more frequent primary care visits, reduced hospital admissions, and shorter inpatient stays associated with ACO models for Medicaid beneficiaries. However, their effectiveness appears to be relatively more limited than that of Medicare ACOs, likely due to variations in state Medicaid programs (35).

Importantly, our findings suggest that the beneficial effect of the CCO model on reductions in ED visits, both all-cause and preventable, dissipates gradually with increasing rurality. Smaller decreases in ED visits among rural CCO enrollees may reflect a limited numbers of providers in rural areas, offering fewer alternatives to EDs for urgent care needs. Moreover, the adoption and performance of CCOs hinge on both financial and non-financial resources. The ACO literature documents that while many infrastructures and configurations are essential for all ACOs to succeed, rural ACOs often face acute shortages of capital and providers (24, 36), making the upfront investments required to develop and operate these innovative models difficult to justify without short-term returns (21). Therefore, financial sustainability efforts such as investments under the CCO model can disproportionately challenge rural providers, potentially influencing the rural-urban differences observed.

Information technology (IT) infrastructure is another big challenge facing rural ACOs. The literature well documents the critical roles of IT infrastructure and health data in ACOs’ success (21–24). Robust electronic health records systems and data-sharing platforms can facilitate better care coordination and targeted management of high-need patients across the continuum of care. However, IT infrastructure remains uneven in rural areas (24), and smaller practices are less likely to implement advanced electronic health systems (37). Despite these challenges, an earlier study shows that some rural ACOs have successfully repurposed existing resources, such as converting a hospital into a community health hub (21), to extend their reach and absorb resources (such as patients, providers, and revenues) from a broader geographic area. Strong IT infrastructure in rural hospitals and clinics can facilitate care coordination and data sharing, which are critical to the success of the CCO model. Therefore, we suggest that CCOs re-think such a strategy that may incentivize rural health care providers to integrate and coordinate care more effectively and thereby benefit their service populations.

While the Oregon Medicaid CCO model was successful in reducing preventable ED visits, this did not translate into a measurable reduction in preventable hospital admissions during our study period. One potential explanation is that preventable hospital admissions were not included among the CCO performance measures, so CCOs lacked direct incentives to reduce preventable hospital admissions. Alternatively, hospital admissions, particularly planned admissions, may be less readily amenable to reduction than ED utilization in Medicaid populations.

At the first glance, the finding of no effect of CCOs on hospital admissions might seem to contradict Yoon et al. (17), which found a substantial reduction in unscheduled, preventable hospital additions among reproductive-age women enrolled in Oregon CCOs. However, these results are not necessarily irreconcilable once we consider differences in populations. Women of reproductive age experience high-volume, ambulatory care-sensitive conditions (e.g., obstetric complications) that are especially responsive to enhanced primary care and care coordination. In contrast, our analysis encompasses the entire Medicaid population—across all ages, comorbidity profiles, and utilization patterns—some of which (e.g., surgical admissions) are less preventable via primary care and care coordination. In a heterogeneous statewide population with lower average preventable-admission rates, the same system-level intervention may yield effects too small to detect statistically. Taken together, our findings and the CCO literature illuminate how both the design of accountable-care arrangements and the characteristics of the populations they serve shape their impact on hospital utilization among Medicaid beneficiaries. Of direct relevance to our analysis, examining rural-urban heterogeneity in the effect of CCOs on preventable hospital admissions represents an essential agenda for future research, as it may shed further light on the mechanisms through which CCOs incentivize providers.

Interpretation of these findings should be considered within the context of several limitations and directions for future research. The effect of the CCO model might spill over into the non-CCO Medicaid beneficiaries, for example, if physicians in a group practice see both CCO and non-CCO patients. Such an effect, if any, would lead our estimates to be attenuated, underestimating the actual effects of CCOs on ED use and hospital admissions. Our data, however, do not provide further information to explore these aspects of providers’ decision-making.

Some Medicaid enrollees were exempt from mandatory CCO enrollment but voluntarily enrolled in CCOs. Potential explanations include an individual's health belief in integrated care, influence by other family members, or personal preferences. This current study lacked data to explore reasons for voluntary CCO enrollment. Nevertheless, our sensitivity analyses, such as dropping dually eligible individuals and running inverse-probability weighted regression, all showed robust results. Future research may include qualitative study components.

Oregon chose not to impose prescriptive organizational strategies on CCOs. They have wide latitude in organizing provider networks, paying providers, and coordinating behavioral and physical health care. This flexibility, designed to allow for innovation and local collaboration, provides the framework for a natural experiment in evaluating different care delivery and quality improvement approaches. Future research could benefit from this unique opportunity to explore heterogeneity among an array of Medicaid ACO models in Oregon and elsewhere. These findings could inform health care delivery transformation for low-income populations in other states.

Our study was limited to utilization outcomes (ED and hospital admissions) and improvements in utilization should ideally translate into better patient outcomes (such as improved health status or reduced mortality) as well as lower costs. Future evaluations of the CCO model should examine direct patient outcomes and cost-effectiveness to fully assess the value of care.

Another noteworthy limitation is that the all-cause hospital admission outcome includes pregnancy-related hospitalizations. While preventable admissions—defined using AHRQ's PQIs—do not include maternity-related hospital stays, all-cause admissions do not make such distinctions. As a result, obstetric admissions (e.g., labor, delivery, or complications thereof) may have diluted the observed effect of the CCO model on all-cause hospitalizations, given that these events are typically less amenable to outpatient prevention strategies. Future research should consider constructing non-pregnancy-related hospitalization measures or conducting subgroup analyses excluding women of childbearing age to better isolate the effects of the accountable care intervention on avoidable hospital use.

We applied PQIs—which are originally designed for assessing population- or area-level performance of ambulatory care—to individual-level claims data. While this approach allows for identification of potentially preventable hospital admissions at the person-month level, PQIs were not intended for evaluating outcomes at the individual level. As such, their application in this context may introduce misclassification or measurement error, and the findings should be interpreted with caution. Future work should consider validation studies or alternative methods more specifically tailored to individual-level preventability.

Although empirically investigating the mechanisms underlying the attenuated CCO effect in rural areas is beyond the scope of our current analysis, future research could leverage administrative database such as ours to elucidate underpinning pathways. For example, linking established indices of community vulnerability (e.g., the CDC Social Vulnerability Index) to medical claims at the enrollee ZIP-code level would allow assessment of whether social determinants, such as poverty and travel distances or drive times to the nearest hospitals, mediate CCO performance. Such analysis will yield a more granular understanding of how community context and spatial barriers influence CCO effectiveness across the rural–urban continuum.

Since its inception, Oregon's Medicaid delivery system transformation through CCOs has emphasized the identification and management of high-cost patients, especially those with severe and chronic behavioral health problems, such as beneficiaries with severe mental illness and comorbid conditions. A further investigation of how CCOs affect frequent health care users will help illuminate mechanisms that incentivize providers’ practices in the networks. As such, the outcomes on rural-urban heterogeneity might differ for other subpopulations of CCO enrollees (e.g., age, race/ethnicity, family income, employment), so examining subpopulation-specific effects could be a valuable extension of this research.

Oregon initiated CCO 2.0 in January 2020, further emphasizing health equity and social determinants of health, such as education, housing, community planning, and transportation barriers, which is especially crucial due to Oregon's predominantly rural and frontier landscape (38). Future research should investigate whether CCO 2.0 better address needs of Medicaid beneficiaries, promoting rural-urban health and health care equity.

## Conclusion

Our findings suggest that Oregon's CCO model achieved meaningful reductions in preventable and all-cause ED visits, though these benefits were less pronounced in rural areas. The diminished impact in rural settings likely reflects persistent structural barriers, such as limited provider capacity and weaker health IT infrastructure. We did not observe a measurable reduction in preventable hospital admissions during the study period; however, this does not necessarily indicate that CCOs failed to influence inpatient care. Rather, such effects may take longer to materialize, may operate through aspects of care not captured by our measures (e.g., care quality or length of stay), or may have been constrained by structural and clinical factors beyond the scope of early CCO incentives.

These findings underscore the importance of context-sensitive ACO design and implementation, particularly in rural environments. As states continue to expand Medicaid ACO models, future research should prioritize understanding rural-urban disparities and refining performance measures to ensure that accountable care arrangements equitably benefit all Medicaid populations.

## Data Availability

The data analyzed in this study is subject to the following licenses/restrictions: the data used for this research are limited for public use under the Data Use Agreement with the Oregon Health Authority. Requests to access these datasets should be directed to Stacy Schubert, stacey.s.schubert@dhsoha.state.or.us.
