# Supplementary material for: Effect of Medicaid accountable care on preventable emergency department and hospital admissions: rural-urban heterogeneity
Source: Front Health Serv. 2025 Jun 26;5:1475140. doi: 10.3389/frhs.2025.1475140 (PMC12240970; doi:10.3389/frhs.2025.1475140)
Supplement: Supplementary file 1 [file Datasheet1.pdf]

Supplemental Materials

Supplemental Figure S1. Trends in ED visits and hospital admissions, 2010-2015

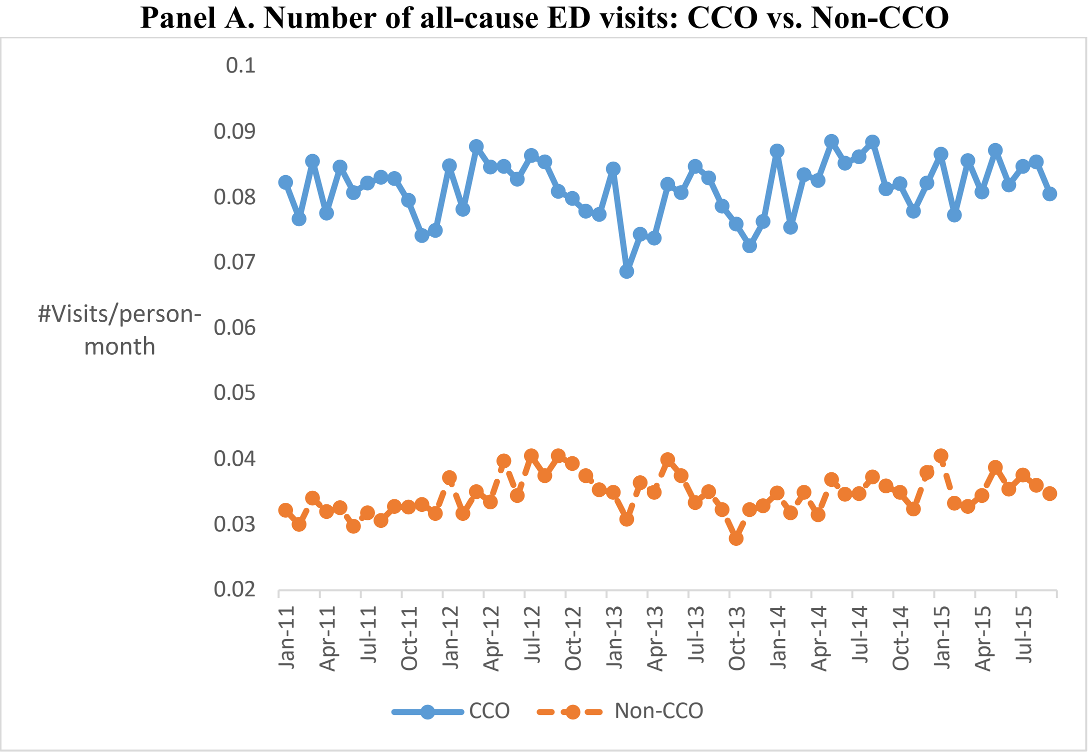

Panel B. Number of preventable ED visits: CCO vs. Non-CCO

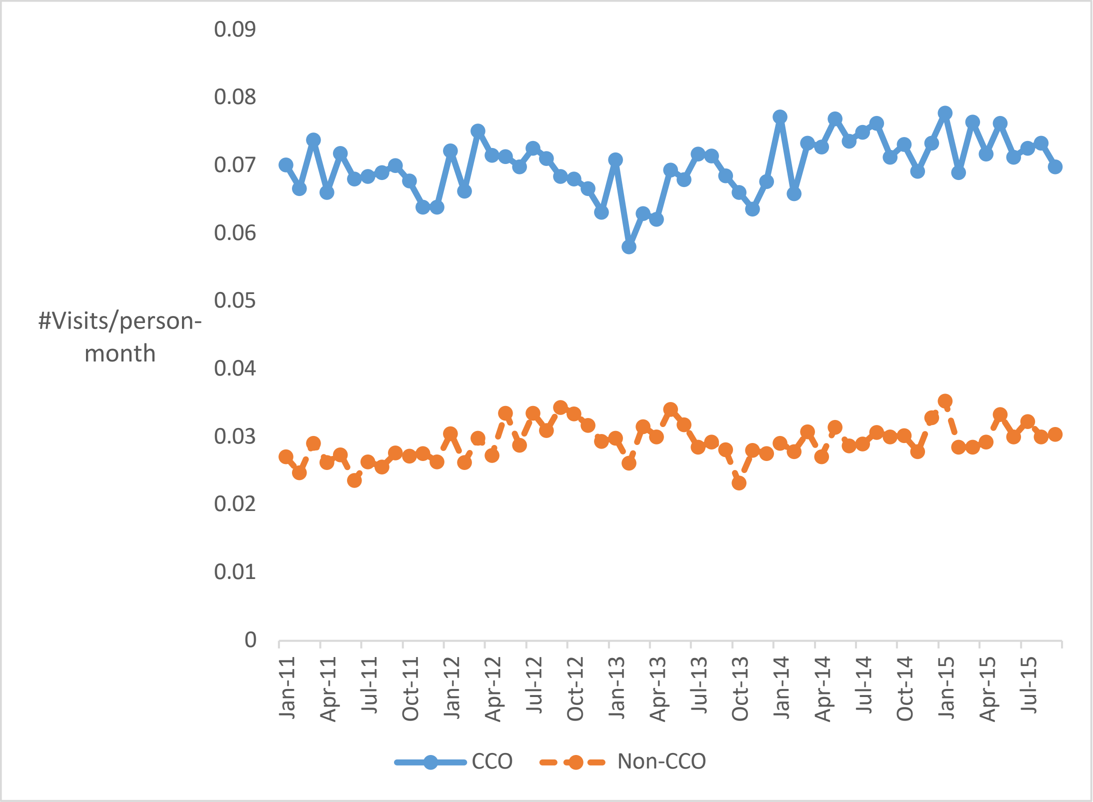

Panel C. All-source hospital admission: CCO vs. Non-CCO

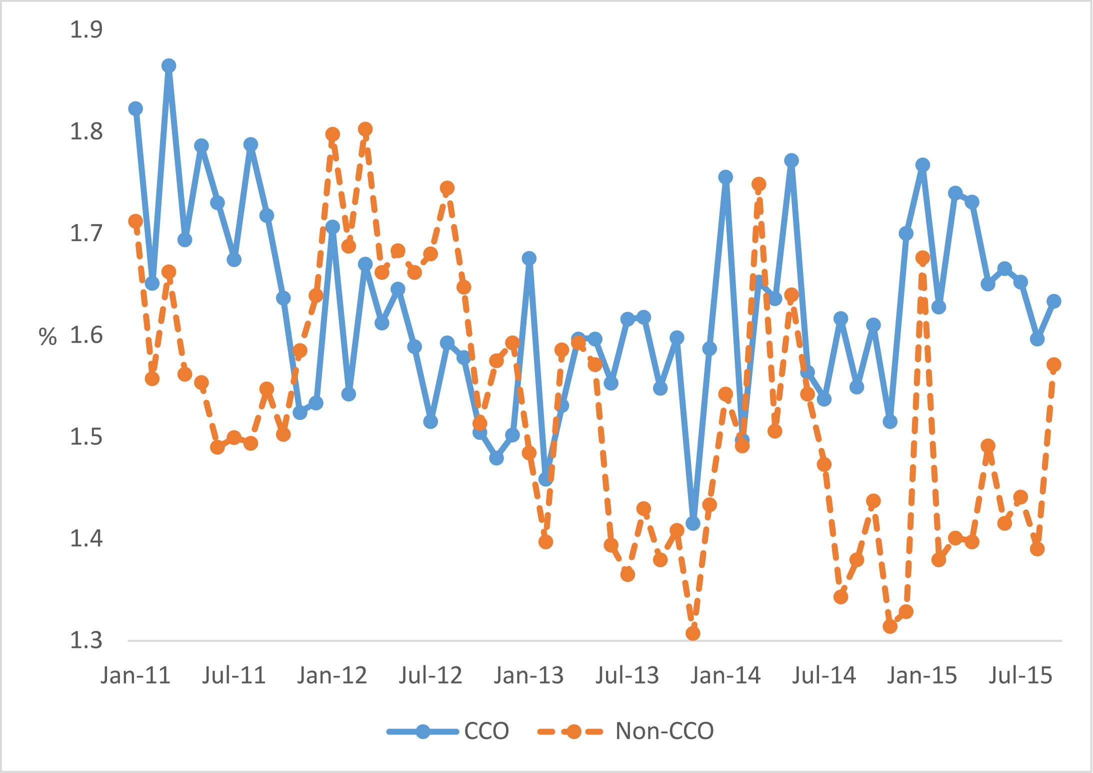

Panel D. Preventable hospital admission: CCO vs. Non-CCO

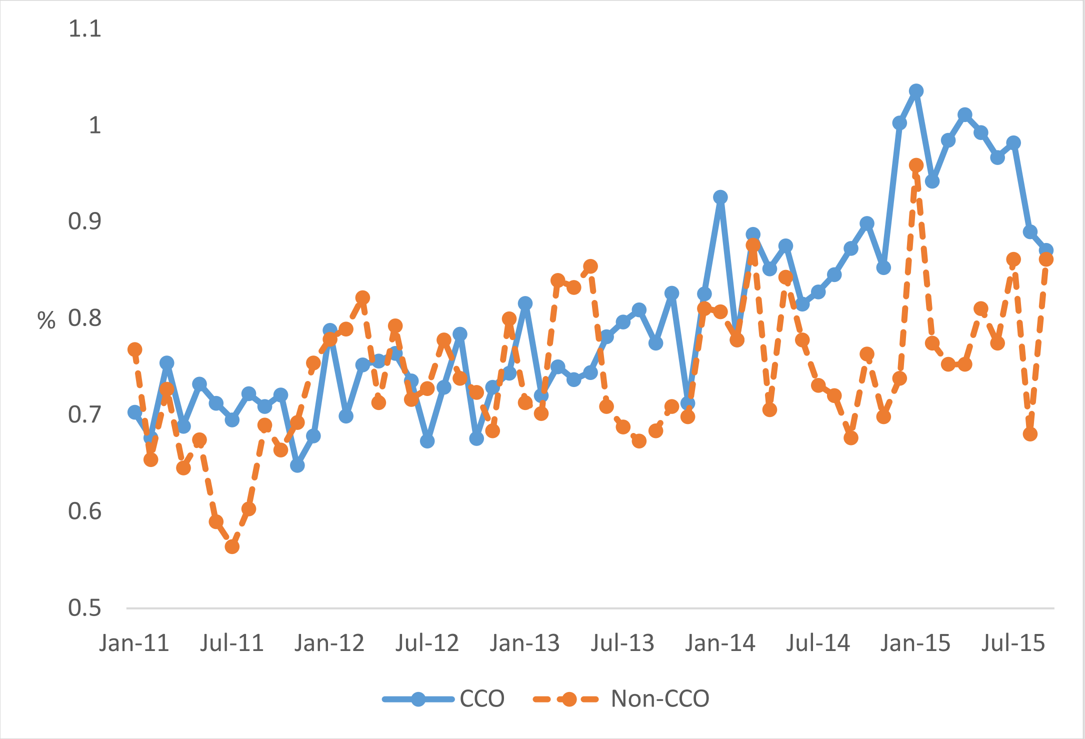

2. Supplemental Tables

Supplemental Table S1. Compositions of program categories for CCO enrollees and non-CCO Medicaid beneficiaries

Program category	CCO enrollees		Non-CCO enrollees	
	n	%	*n*	%
TANF	1,506,060	20.3	29,888	1.51
Pregnancy	98,920	1.33	2,581	0.13
Aid to the Blind/Aid to the Disabled	2,593,832	34.9	635,235	32.0
Expansion[a]	1,634,389	22.0	26,567	1.34
Dual eligibility	360,321	4.85	497,954	25.1
Cancer	12,781	0.17	864	0.04
Other programs	31,049	0.42	472,613	23.82
Uncategorized	1,194,587	16.1	316,626	16.0
Total	7,431,939	100	1,982,328	100%

Note: CCO = Coordinated Care Organization. TANF=Temporary Assistance for Needy Family.
[a]Medicaid coverage for adults made newly enrolled in Oregon's Medicaid beginning in January 2014 under the Affordable Care Act.

Supplemental Table S2. Frequencies of all-cause and preventable ED visits

Number of visits per month	All-cause ED visits		Preventable ED visits	
	Frequency	%	Frequency	%
0	8,910,491	94.6	8,988,263	95.5
1	399,993	4.25	345,440	3.67
2	74,571	0.79	59,285	0.63
3	18,614	0.20	13,969	0.15
4	6,058	0.06	4,331	0.05
5	2,259	0.02	1,543	0.02
6	1,051	0.01	658	0.01
7	515	0.01	335	0.004
8	286	0.003	188	0.002
9	161	0.002	92	0.001
10	94	0.001	54	0.001
11	54	0.001	45	0.0005
12	47	0.0005	23	0.0002
13	24	0.0003		
14	8	0.0001		
Total	9,414,226	100	9,414,226	100

Note: ED = Emergency department.

Supplemental Table S3. Falsification regression analysis for parallel trend assumption

Robustness test & main variables	ED visit		Hospital admission	
	All-cause	Preventable	All-cause	Preventable
Falsification test 1[a] (N=4,702,558)				
CCO×Post	–0.0280	–0.0172	–0.0909	–0.0563
	(0.0223)	(0.0239)	(0.0694)	(0.0573)
Falsification test 2[b] (N=4,702,558)				
CCO×Post	–0.0186	–0.0091	0.0545	0.0203
	(0.0170)	(0.0133)	(0.0394)	(0.0569)

Note: Pooled negative binomial models on time-demeaned regressors were estimated for the ED visit outcomes because panel count data models do not allow for inverse–probability weighting. Cluster-robust standard errors were obtained. For the hospital admission outcomes, fixed–effects linear probability models were estimated and standard errors in parentheses were corrected for clustering on individuals.

[a]Reported are (average) marginal effects of a false CCO implementation period (which equals 1 for years 2011 and 2012). The study period was restricted to the pre-CCO period of 2010 through 2012.

[b]Reported are (average) marginal effects of a false CCO implementation period (which equals 1 for year 2012). The study period was restricted to the pre-CCO period of 2010 through 2012.

Supplemental Table S4. Propensity–score logit model of CCO enrollment

Variables	Coefficient
Female	0.4022***
	(0.0018)
Hispanic	–1.6986***
	(0.0031)
Race (Reference: White)	
Black	0.8753***
	(0.0210)
AIAN	–1.6917***
	(0.0179)
Asian	–0.7643***
	(0.0248)
NHPI	–1.8580***
	(0.0527)
Unknown/missing	–1.4805***
	(0.0080)
Age	–0.0408***
	(0.0001)
Rurality (Reference: Urban)	
Large Rural	0.1710***
	(0.0091)
Small Rural	–0.4607***
	(0.0152)
Race–Age Interactions	
Black × Age	–0.0107***
	(0.0004)
AIAN × Age	0.0151***
	(0.0004)
Asian × Age	0.0286***
	(0.0006)
NHPI × Age	0.0231***
	(0.0013)
Unknown/missing × Age	0.0346***
	(0.0002)
Rurality–Age Interactions	
Large rural × Age	–0.0086***
	(0.0002)
Small rural × Age	0.0009**
	(0.0003)
Constant	3.1354***
	(0.0046)
N	9,413,327

Note: CCO = Coordinated Care Organization. AIAN = American Indian/Alaska Native. NHPI: Native Hawaiian/Pacific Islander. Standard errors in parentheses are corrected for clustering on individuals.

$^{**}p < .01$.
$^{***}p < .001$.

Supplemental Table S5. Heterogeneous effects of CCO on ED visits and hospital admissions by rurality of residence location: Fixed-effects negative binomial and fixed-effects logit coefficients

Variable	Number of ED visits		Hospital admission	
	All-cause	Preventable	All-cause	Preventable
CCO×Post (for urban)	–0.3533***	–0.3535***	–0.0374*	–0.0845***
	(0.0165)	(0.0176)	(0.0166)	(0.0235)
CCO×Post×Large rural	–0.0902***	–0.0888***	0.0092	0.0506
	(0.0109)	(0.0117)	(0.0286)	(0.0404)
CCO×Post×Small rural	0.0471*	0.0278	–0.1363*	–0.1593*
	(0.0230)	(0.0248)	(0.0535)	(0.0738)
Large rural	–0.2587***	–0.2899***	–0.1688***	–0.2054***
	(0.0099)	(0.0107)	(0.0416)	(0.0604)
Small rural	–0.5229***	–0.5533***	–0.2164***	–0.2826**
	(0.0182)	(0.0198)	(0.0585)	(0.0877)
Post	0.1593***	0.2813***	0.0024	0.0638
	(0.0157)	(0.0173)	(0.0290)	(0.0407)
Age	–0.0058***	–0.0046***	–0.0095	0.0082
	(0.0003)	(0.0003)	(0.0098)	(0.0142)
Time trend	–0.0050***	–0.0105***	–0.0006	–0.0013
	(0.0003)	(0.0004)	(0.0011)	(0.0015)
CCO×Time trend	0.0118***	0.0123***	0.0021*	0.0033**
	(0.0003)	(0.0004)	(0.0008)	(0.0011)
N	9,414,226	9,414,226	4,232,338	4,222,291

Note: CCO = Coordinated Care Organization. ED = Emergency department. All models are fully specified but only main coefficients are reported. Fixed-effects negative binomial models were used for the count ED visit outcomes. Conditional logit models were estimated for the binary hospital admission outcomes. Standard errors in parentheses are corrected for clustering on individuals. The effect of the CCO model on ED visits also appears to differ by rurality of residence location. The coefficient on the CCO×Post variable is negative and statistically significant for the ED visit outcomes, which may imply that the CCO model is associated with decreases in both all-cause and preventable ED visits for urban residents. Statistically significant but smaller negative coefficients on the triple interaction term were found for Medicaid enrollees in large rural areas. The coefficients on the triple interaction term for Medicaid enrollees in small rural areas were positive. However, linear combination of coefficients indicates negative coefficients for residents in small rural areas. The main coefficient on the triple interaction term for the hospital admission outcomes was negative and statistically significant for urban and small rural areas. It was positive and insignificant for hospital admission. However, linear combination of coefficients indicates negative coefficients for residents in large rural areas.

$^{*}p < .05$.

$^{**}p < .01$.

$^{***}p < .001$.

Supplemental Table S6. Robustness checks

Robustness test & main variables	ED visit		Hospital admission	
	All-cause	Preventable	All-cause	Preventable
Panel A: Overall effect of CCO				
100% on Medicaid (N = 1,438,259)				
CCO×Post	–0.9599***	–1.0012***	0.0307	0.0260
	(0.0458)	(0.0505)	(0.1509)	(0.2193)
60% on Medicaid (N =13,498,036)				
CCO×Post	–0.3538***	–0.3636***	–0.0412	–0.0005
	(0.0162)	(0.0176)	(0.0318)	(0.0450)
Not inverse probability weighted				
CCO×Post	–0.3556***	–0.3488***	–0.0248	–0.0062
	(0.0412)	(0.0452)	(0.0387)	(0.0525)
Drop TANF beneficiaries (N=4,586,120)				
CCO×Post	–0.9599***	–1.0012***	–0.0829	0.1851
	(0.0458)	(0.0505)	(0.0591)	(0.1468)
Drop dually eligible beneficiaries (N=9,160,934)				
CCO×Post	–0.3421***	–0.3453***	–0.0376	0.0097
	(0.0164)	(0.0180)	(0.0323)	(0.0462)
Drop expansion population (N=9,390,018)				
CCO×Post	–0.3419***	–0.3450***	–0.0402	–0.0001
	(0.0164)	(0.0180)	(0.0317)	(0.0450)
Panel B: Effect of CCO by rurality of residence location				
100% on Medicaid				
CCO×Post (for urban)	–0.8585***	–0.8963***	0.0509	0.0314
	(0.0457)	(0.0504)	(0.1513)	(0.2197)
CCO×Post×Large rural	–0.0850***	–0.0751***	–0.0779	–0.0180
	(0.0155)	(0.0168)	(0.0461)	(0.0652)
CCO×Post×Small rural	0.0525	0.0393	–0.1001	–0.0415
	(0.0331)	(0.0362)	(0.1100)	(0.1367)
60% on Medicaid				
CCO×Post (for urban)	–0.3088***	–0.3144***	–0.0328	–0.0032
	(0.0165)	(0.0181)	(0.0320)	(0.0453)
CCO×Post×Large rural	–0.0929***	–0.0899***	–0.0397	0.0440
	(0.0099)	(0.0108)	(0.0240)	(0.0359)
CCO×Post×Small rural	0.0344	0.0246	–0.1096*	–0.0749
	(0.0208)	(0.0230)	(0.0442)	(0.0678)
Not inverse probability weighted				
CCO×Post (for urban)	–0.3268***	–0.3233***	–0.0172	–0.0072
	(0.0408)	(0.0448)	(0.0388)	(0.0527)
CCO×Post×Large rural	–0.1781***	–0.1703***	–0.0300	0.0392
	(0.0396)	(0.0420)	(0.0257)	(0.0373)
CCO×Post×Small rural	–0.1440*	–0.1418	–0.1208*	–0.1097

	(0.0706)	(0.0755)	(0.0498)	(0.0697)
Drop TANF beneficiaries (N=4,586,120)				
CCO×Post (for urban)	–0.8585***	–0.8963***	–0.0660	0.1781
	(0.0457)	(0.0504)	(0.0593)	(0.1472)
CCO×Post×Large rural	–0.0850***	–0.0751***	–0.1169***	0.0631
	(0.0155)	(0.0168)	(0.0331)	(0.0692)
CCO×Post×Small rural	0.0525	0.0393	–0.0768	–0.0445
	(0.0331)	(0.0362)	(0.0571)	(0.1140)
Drop dually eligible beneficiaries (N=9,160,934)				
CCO×Post (for urban)	–0.3216***	–0.3309***	–0.0291	0.0071
	(0.0168)	(0.0184)	(0.0325)	(0.0465)
CCO×Post×Large rural	–0.0963***	–0.0930***	–0.0439	0.0366
	(0.0101)	(0.0111)	(0.0250)	(0.0384)
CCO×Post×Small rural	0.0372	0.0261	–0.0991*	–0.0487
	(0.0213)	(0.0235)	(0.0455)	(0.0717)
Drop expansion population (N=9,390,018)				
CCO×Post (for urban)	–0.3087***	–0.3140***	–0.0316	–0.0028
	(0.0165)	(0.0181)	(0.0319)	(0.0453)
CCO×Post×Large rural	–0.0928***	–0.0895***	–0.0417	0.0433
	(0.0099)	(0.0108)	(0.0240)	(0.0359)
CCO×Post×Small rural	0.0337	0.0237	–0.1117*	–0.0764
	(0.0208)	(0.0230)	(0.0442)	(0.0679)

Note: Coefficients are reported unless noted otherwise. Pooled negative binomial models on time-demeaned regressors were estimated for the ED visit outcomes because panel count data models do not allow for inverse–probability weighting. Cluster-robust standard errors were obtained. For the hospital admission outcomes, fixed–effects linear probability models were estimated and standard errors in parentheses were corrected for clustering on individuals.

*$p < .05$.

**$p < .01$.

***$p < .001$.
